# Bacterial Ghosts-Based Vaccine and Drug Delivery Systems

**DOI:** 10.3390/pharmaceutics13111892

**Published:** 2021-11-08

**Authors:** Haojie Chen, Hao Ji, Xiangjun Kong, Pengyu Lei, Qinsi Yang, Wei Wu, Libo Jin, Da Sun

**Affiliations:** 1 Institute of Life Sciences & Engineering Laboratory of Zhejiang Province for Pharmaceutical Development of Growth Factors, Wenzhou University, Wenzhou 325035, China; haojiechen@stu.wzu.edu.cn (H.C.); 20180031@wzu.edu.cn (H.J.); xiangjunkong@um.edu.mo (X.K.); 20210155@wzu.edu.cn (P.L.); david2015@cqu.edu.cn (W.W.); 2 State Key Laboratory of Quality Research in Chinese Medicine, Institute of Chinese Medical Sciences, University of Macau, Macao 999078, China; 3 Wenzhou Institute, University of Chinese Academy of Sciences, Wenzhou 325000, China; yangqs@wiucas.ac.cn; 4 Key Laboratory for Biorheological Science and Technology of Ministry of Education & State and Local Joint Engineering Laboratory for Vascular Implants, Bioengineering College of Chongqing University, Chongqing 400030, China

**Keywords:** bacterial ghosts, gene E, vaccines, immune, delivery system

## Abstract

Bacterial ghosts (BGs) are empty bacterial envelopes of Gram-negative bacteria produced by controlled expressions of cloned gene E, forming a lysis tunnel structure within the envelope of the living bacteria. Globally, BGs have been used as vaccine delivery systems and vaccine adjuvants. There is an increasing interest in the development of novel delivery systems that are based on BGs for biomedical applications. Due to intact reservation of bacterial cell membranes, BGs have an inherent immunogenicity, which enables targeted drug delivery and controlled release. As carrier vehicles, BGs protect drugs from interference by external factors. In recent years, there has been an increasing interest in BG-based delivery systems against tumors, inflammation, and infection, among others. Herein, we reviewed the preparation methods for BGs, interactions between BGs and the host, and further highlighted research progress in BG development.

## 1. Introduction

Delivery systems for drugs, nucleic acids, and other biomolecules are recent developments in biotechnology [1,2]. There is a growing interest in the design and development of novel targeted delivery systems. Bacterial derivatives, including bacterial ghosts (BGs), extracellular vesicles, and alimental toxins, are popular biological nanomaterials that are potential vaccine and drug carriers [3]. These delivery platforms retain many of the advantages of bacteria, including the ability to colonize and target human tissues, enhance immunogenicity of vaccines, and have good loading capacities. Advances in genetic engineering and chemical biotechnology have facilitated the development of different types of BGs, which will be important in immobilized enzyme technology, agriculture and medicine [4]. Recently, BGs have received increased attention as potential candidates for targeted delivery of biomolecules [5,6].

**Figure 1:**
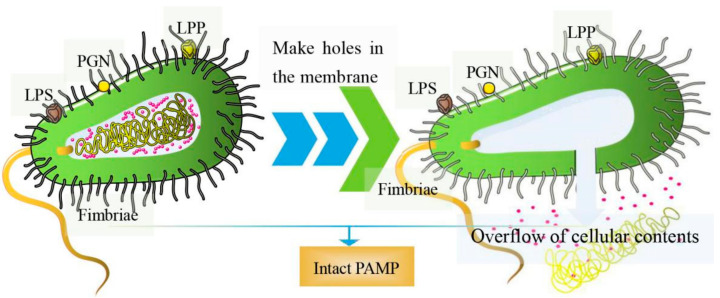
The diagram of BGs. (**left**) Pores are formed on the surface of bacteria; (**right**) overflow of cellular contents including nucleic acids, ribosomes, and other components.

Essentially, BGs are bacterial shells with pores. Genetic engineering or chemical methods can be used to induce the release of cellular contents; hence, they have no nucleic acids, ribosomes, or other components [7]. As such, the structural integrity of surface antigens on most BGs remains intact [8]. The preparation of inactivated vaccines using methods such as formaldehyde and heat treatments can destroy the surface structures of the bacteria [9,10]. Contrarily, BGs prepared by genetic engineering retain all the structural antigens expressed by pathogenic bacteria, which induces very strong, effective humoral and cellular immune responses [11]. The structure of BGs is shown in Figure 1. They contain pathogen-associated molecular patterns (PAMPs), such as lipopolysaccharide (LPS), lipoprotein (LPP), peptidoglycan (PGN), and fimbriae, among others, which are highly conserved structures on the outer cell bacterial wall [12]. Once in the host, BGs are recognized by pattern recognition receptors (PRR) on immune cells, stimulating the production of several immune mediators that induce the maturation of antigen-presenting cells (APCs), such as dendritic cells (DCs) [13,14]. In general, pathogenic bacterial ghost serotypes are well preserved, and high concentrations of BGs can provide a high immunogenicity. Moreover, they can only be purified by washing, centrifugation and freeze-drying [15]. Based on these novel biological characteristics, BGs are potential vaccine delivery systems [16]. They have been progressively adopted in the delivery of nucleic acids, proteins, and chemical drugs [17].

BGs are applied in several disciplines including biology, chemistry, pharmacy, biomedicine, and material sciences, among others [18]. In this paper, we reviewed the methods for BG preparation and how they induce immunity in hosts. Moreover, we reviewed the principles for designing BG-based delivery systems as well as the advantages and limitations of BGs in biomedical applications. Finally, we discussed the administration routes for BG-based delivery systems, particularly the oral-digestive route.

## 2. Methods for BGs Preparation

### 2.1. Genetic Engineering

**Figure 2:**
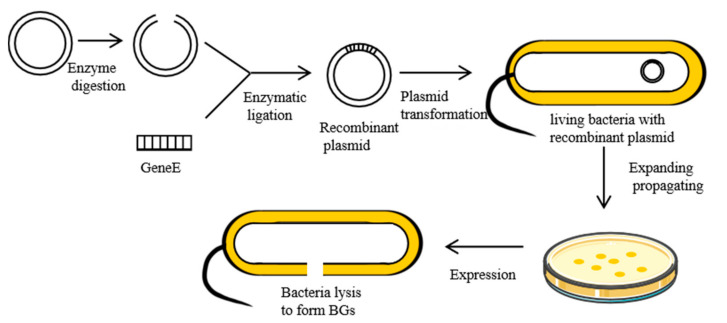
Preparation of the BGs using genetic engineering.

When ΦX174, a single-stranded DNA phage, infects Gram-negative bacteria, it inhibits the activities of the enzyme phospho-MurNAc-pentapeptide translocase (MraY) on the bacteria membrane. This is mediated by lysis protein E, a hydrophobic protein that inhibits the synthesis of PGN in bacterial cell walls [19]. First, the hydrophobic N-terminal binds the inner membrane of the bacterial cell wall. Then, the conformation of protein E changes, binding its hydrophobic C-terminal across the inner and periplasmic spaces to the outer membrane of the cell wall [20]. This disruption causes secondary effects, such as activation of phosphatase activities, and increased membrane mobilities. This increases internal osmotic pressures, inducing the release of cell contents [21]. Currently, the most commonly used method for the preparation of BGs involves cloning lysis gene E (276 bp, accession number: MF426914.1) into expression regulation systems of Gram-negative bacteria, which utilizes the switch function of the control element to control expression [22]. The preparation process for BGs is shown in Figure 2.

Constructed BGs are about 1–2 μm long and 0.5–2 μm wide. Lysis protein E connects the inner and outer membranes, minimizing the loss of enzymes in periplasmic spaces. During bacterial lysis, inner and outer membrane structures remain intact. Diameters of transmembrane pores are about 40–200 nm, but, under osmotic pressure, they can reach 500 nm [23]. Pore sizes are determined by sieve sizes of the PGN layer in the cell wall [24].

Transmembrane tunnels are formed at the bacterial division site or polar cap of bacteria, but mostly at the bacterial site, which is related to Z-ring formation mechanisms during bacterial division. The bacterial division protein FtsZ is necessary for Z-ring formation [25]. Witte, A. et al. found that protein E-mediated lysis occurs in the presence of GTPase activities of FtsZ, which is independent of Z-ring structure [26]. However, the mechanisms involved in conformational changes of protein E, which are caused by GTPase activities of FtsZ, should be further investigated.

Expressions of temperature-sensitive λ pL/pR-cI857 or chemically induced expression systems (efficient expression systems of all kinds of hybrids based on Lac promoter) are generally used as markers for selection of successful transformation [23]. For λ pL/pR-cI857 expression, at appropriate temperatures, bacteria are cultured to the logarithmic growth phase, before raising the temperature to 42 °C to induce the expressions of gene E [27,28,29]. For chemically induced expression systems, IPTG or arabinose is added to the original culture medium to obtain BGs [21]. Obtained BGs are lyophilized. Lyophilized BGs can be stored at room temperature for many years [30]. Barisani Asenbauer et al. cultured *E. coli* in a fermentor until OD600 = 0.9, which took about 90 min. Under temperature-sensitive expression systems, a temperature of 42 °C induces the expressions of protein E. After 120 min of expression, lytic efficiency reached 99.9% [31]. Langemann et al. prepared BGs by heating a mixed culture media of bacteria to 42 °C. After 2 h, bacterial lytic efficiency reached 99.99% [21]. Even with the excellent lysis, residual pathogenic bacteria are potentially harmful. Therefore, secondary inactivation is required to achieve complete lysis. Since UV radiation or formaldehyde inactivation can damage the structures of BG surface antigens, the commonly used method involves the addition of β-acetone (BPL) for inactivation, freeze drying, and storage at −20 °C [10,32]. Gentamicin and streptomycin are also used to inactivate non-lysed bacteria [33]. Double gene inactivation has also been used for secondary inactivation. Zhu et al. enhanced the lytic efficiency of bacteria by incorporating the staphylococcal nuclease A(SNUC) gene into a lysis plasmid (mE-L-SNA) expressing the E fusion gene. The lytic efficiency of *E. coli* at the logarithmic growth stage reached 99.99995%, thus improving the safety of BGs [34]. Tian et al. constructed *Streptococcus pullorum* ghosts by fusing the antimicrobial peptide gene, SMAP29, with lysis gene E. Twenty-four hours after induced lysis, they did not find any viable bacteria [35]. Hjelm et al. constructed a mutant strain of *E. coli* (MC4100) by deleting the ASD gene, which encodes aspartate semialdehyde dehydrogenase involved in diaminopimelic acid (DAP) synthesis. The growth of mutant strains is entirely dependent on the amount of DAP added to the culture medium. After gene E was induced, the target bacteria/BGs were incubated in LB medium without DAP for 12 h, centrifuged, collected, and freeze-dried to completely inactivate *E. coli* [36]. Chemical and genetic engineering methods can inactivate residual pathogenic bacteria.

Currently, regulation of the expression of the gene E lysis system has been successfully applied against several gram-negative bacteria strains, *Salmonella typhimurium* [37], *Salmonella enteritidis* [8], *Vibrio cholerae* [38], *Pectobacterium cypripedii* [39], *Helicobacter pylori* [40], *Actinobacillus-pleuropneumoniae* [41], *Haemophilus influenzae* [42], *Pasteurella multocida* [43], *Brucella* [44], and *Aeromonas hydrophila* [45], among others. Given the wide spectrum of bacteria, these findings imply that any suitable gene E carrier, perhaps all Gram-negative bacteria, can produce lysis-based BGs. However, the preparation methods for BGs are associated with several challenges. (I) Plasmids are not applicable to all Gram-negative bacteria, which should be investigated and modified for each strain. Uneven distributions of plasmids during bacterial division leads to plasmid loss; therefore, there is a need to determine whether gene E can be cloned into the bacterial genome. (II) During the preparation of BGs, the antibiotic resistance gene is introduced alongside lysis gene E. The antibiotic resistance gene can be laterally transferred in the environment [46]. (III) There are many lytic resistant mutants, especially *E*. *coli*, which requires double gene inactivation, such as combining gene E and gene SNUC [47].

Gens other than gene E can also be used to prepare BGs. For instance, Ronchel et al. prepared *Pseudomonas putida*-based BGs by cloning heterologous GEF proteins in these bacteria [48]. BGs have also been prepared by cloning plasmid pDKL02 expressing lysis genes S, R, and Rz from bacteriophage λ into *E. coli*, *Acinetobacter calcoacetate* and *Pseudomonas stephensi* [49]. At the same time, they compared the gene E-mediated lysis system with this gene lysis system. They found that the nucleic acid produced by the gene E-mediated lysis system could be completely degraded after 1 h in vitro, which was more efficient for the preparation of adjuvants.

### 2.2. The Chemical Method

**Figure 3:**
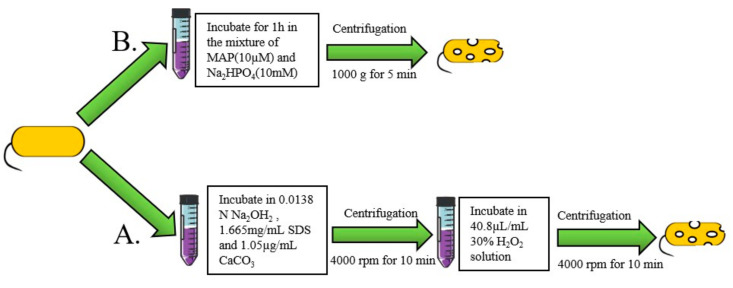
Chemical method for preparation of BGs. (**A**) Preparation of BGs using the model amphiphilic peptide (MAP). (**B**) The two-step method for preparation of BGs.

The “sponge-like” method is a common chemical process for preparing BGs. In this method, pores are made through bacterial cell walls using chemical reagents. Then, cellular contents are removed by centrifugation (Figure 3A). Amara et al. used less than the minimum inhibitory concentration (MIC) of chemicals such as NaOH, SDS, H_2_O_2_, and CaCO_3_ to prepare BGs. Then, they used the Plackett–Burman experimental design to optimize the preparation conditions for “sponge-like” *E. coli* ghosts [50]. Sheweita et al. prepared “sponge” BGs by incubating *Acinetobacter baumannii Ali190* in a mixture of NaOH, Na_2_CO_3_ and a solution of H_2_O_2_ [11]. Under these methods, cell wall integrity remains intact. Sameh et al. designed a novel chemical method of preparing BGs by culturing *Salmonella* for 24 h in a culture media supplemented with 7% Tween 80 [51]. The pH of the medium was reduced to 3.6 using lactic acid. Tween 80 causes the dissolution of hydrophobic components in the outer membrane of the bacteria, thus forming weak areas. These areas facilitate puncture formation caused by the sudden decrease in pH. In another biochemical method, BGs were developed by incubating bacteria in an artificial synthetic model amphiphilic peptide (MAP) dissolved in Na_2_HPO_4_ solution (Figure 3B) [52,53]. The chemical process can be performed at any stage of bacterial growth and only requires dilution to control OD_600_ = 0.1. The efficiency of gene E-mediated lysis was best in the logarithmic growth phase, and lysis in the stationary phase resulted in the survival of live bacteria [54]. Furthermore, the chemical method is not limited to Gram-negative bacteria as it is also effective for Gram-positive bacteria and yeasts [55].

Rapid preparation of BGs using chemical agents eliminates the limitations associated with the use of lysis gene E, particularly genetic restriction-modification [2]. The chemical method is simple, rapid, and does not change the three-dimensional morphology of cells, except for producing holes. However, chemicals may denature surface immunogenic antigens. Excess holes may disrupt the controlled release property. Follow-up experiments should be performed to solve these challenges. Therefore, genetic engineering methods are still the most widely used techniques.

## 3. The Association between BGs and Target Cells

**Figure 4:**
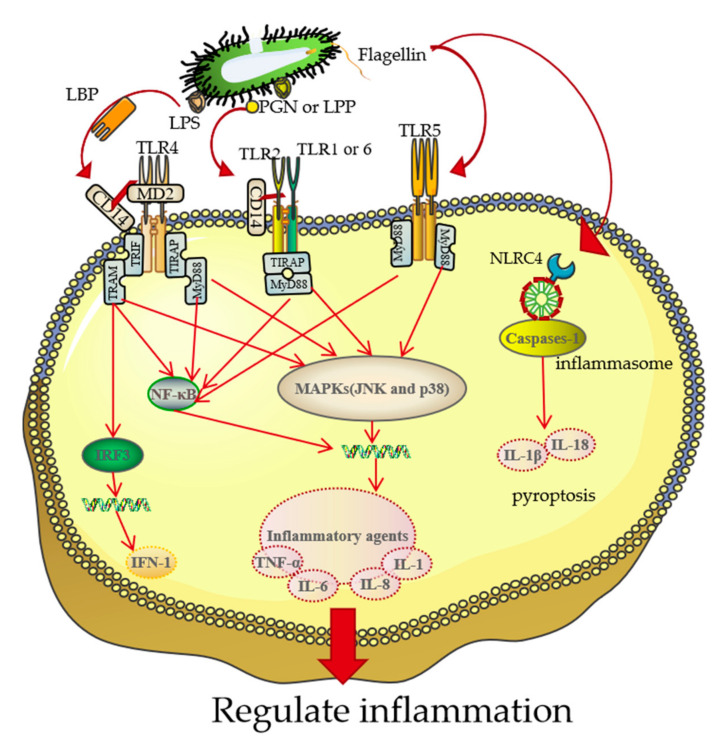
The pathway that mediates binding of BGs to TLRs on immune or epithelial cells. The cytoplasmic portion of TLR contains a *Toll*/IL-1 receptor homolog domain that induces the recruitment of various adapters such as MyD88, TRIF, TIRAP/MAL, and TRAM that regulates specific biological responses [57,58]. Membrane CD- 14 mediates binding between LPS on BGs with a TLR2 homologous dimer, activating the MyD88-independent pathway to produce IFN-1 [59]. CD-14 also mediates binding of surface LPP and PGN with TLR2/TLR6 or TLR2/TLR1 heterodimers, activating the MyD88-dependent pathway. The MyD88-independent and MyD88-dependent pathways eventually stimulate the production of IFN-1 and NF-κB, which activate the secretion of inflammatory factors such as IL-1β, IL-8, and TNF-α [4]. These cytokines induce and regulate inflammation, recruit immune cells and induce apoptosis. In addition to activating the TLR5 pathway and the subsequent MAPK pathway, flagella can also activate NLRC4 inflammasome pathway in cells, which induces cell scorch death.

The intensity of immune responses depends on the physical and chemical properties of the antigen and host immune systems [56]. As vaccine or vaccine adjuvants, BGs can enhance host innate immune responses to antigens. BGs possess PAMPs because they are Gram-negative bacterial shells without internal nucleic acids [29]. Therefore, this section focuses on effects of Gram-negative bacterial PAMPs, such as flagella, LPS, and PGN on the immune system (Figure 4).

LPS can induce the immune response mediated by *Toll*-like receptor 4 (TLR4) [60,61], which is mainly distributed on the surfaces of APCs. BGs stimulate APCs through the binding of LPS on BGs to TLR4 on these cells. There are two intracellular signaling pathways downstream of TLR4: MyD88-dependent and MyD88-independent pathways. The MyD88-dependent intracellular signaling pathway involves activation of the NF-κB-mediated inflammatory factor gene expression (TNF-α, IL-1,6,8, etc.) and activation of the MAPK pathway (including JNK and P38 pathways) [62,63]. The MyD88-independent pathway (TRIF dependent signaling pathways) induces the expressions of transcription factor IRF3, resulting in expressions of type I interferon (IFN-1) and activation of NF-κB to promote the expressions of inflammatory factors. Activation of the TLR4 receptor promotes the maturation of DCs and differentiation of T cells into Th1 cells [64]. In conclusion, LPS on BGs enhances innate and adaptive immune system responses.

The bacterial flagellum is an adhesion structure that mediates host cell colonization. Given their antigenic properties, flagella are ideal adjuvants and vaccine candidates. *Clostridium difficile* [65] and polymeric flagellin filaments are examples of flagella-based vaccines [66]. The flagellum structure on BGs remains intact [31]. Flagellin can induce the expressions of TLR5 and *Nod*-like receptor card domain containing 4 (NLRC4) inflammasomes’ signaling pathways [67,68]. TLR5 is mainly distributed on the surface of intestinal epithelial cells. The binding of flagellin to TLR5 activates the MyD88-dependent signaling pathway and induces NF-κB-mediated inflammatory responses [69]. NLR4 forms NLRC4 inflammasomes and induces the recruitment as well as activation of caspase-1 upon detecting flagellin in the cytoplasm. Activated caspase-1 hydrolyzes, stimulates, and induces the maturation and release of pro-IL-18 and pro-IL-1β to participate in the immune responses [61,70].

*Toll*-like receptor 2 (TLR2) on the surface of macrophages and DCs mainly recognizes PAMPs such as PGN and LPP of BGs [71]. Lysozymes secreted by epidermal cells break down PGN on BGs, then mediate its binding to corresponding receptors. The binding of PGN to TLR1/2 stimulates the expression of NF-κB via the MyD88-dependent pathway, which is involved in adaptive immune responses [72,73]. In addition, binding of the PGN fragment to the NLR1/2 receptor induces the production of TNFα IL-1 and IL-6 [74]. The binding of LPP to TLR2/6 activates pathways similar to those activated by PGN, and subsequent production of TNF-α as well as inflammatory factors, such as IL-6 and macrophage inflammatory protein 2 [75].

Not only do BGs bind APCs and epithelial cell surface receptors to cause immune responses, they can also be internalized by these cells, such as human conjunctiva-derived epithelial cells (HCDECs), Caco-2, etc. [17]. Kudela et al. revealed that independent of the bacterial species, there were no cytotoxic effects of BGs on HCDECs, and HCDECs strongly internalized BGs [30,76]. BGs are a natural adjuvant that can induce a strong antigenic cross-presentation when loaded with DNA or proteins [77]. In an in vitro DCs experiment, Michalek et al. found that administration of BGs loaded with the autologous tumor lysate activated both MHC-I and MHC-II antigen processing and presentation pathways, promoting the proliferation of CD4+ and CD8+ T cells [12]. DC immunotherapy stimulates DC maturation and cross-presentation through BG surface polyantigenicity. After sticking to the surface of targeted areas, BGs, or their released contents, could be endocytosed by target cells. BGs promote antigen presentation, including MHC-I and MHC-II by stimulating DC maturation. There are two theories on the presentation of MHC-I in DCs: (I) the DC engulfs antigenic substances into the intracellular body through endocytosis, and then “somehow” flows out into the cytoplasm, where it forms a pathway to merge with the classical MHC-I antigen complex; (II) DCs phagocytose antigenic substances into the intracellular body through endocytosis, and then fuse directly with the intracellular body carrying no-load MHC-I through membrane vesicles.

As mentioned earlier, adhesion factors on the bacterial surface, LPS, PGN, and other immune stimulants on BGs remain intact. As such, they are well recognized by APCs in different tissues. Studies have shown that BG-based vaccines are more effective than inactivated bacterial vaccines. Xuhua Ran et al. immunized mice with a *Pasteurella* *multocida* (PM) ghost [43]. Compared to inactivated PM, the BGs group produced more lymphocytes, more IL-4 and γ-INF, and had a better defense against PM. Moreover, BGs protect internal molecules from degradation; are useful for targeted delivery of nucleic acids, proteins, and chemical drugs; and their contents are slowly delivered by adhesion to cells [14,78]. The various applications of BGs are shown in Table 1.

## 4. BGs-Based Vaccine

### 4.1. DNA Vaccines

Naked DNA vaccines are limited by their weak immunogenicity. To elicit stronger immune response, they are usually combined with various adjuvants, such as alum. However, the currently used adjuvants have various limitations and the adjuvant options available to any recombinant vaccine manufacturer are very limited. However, carrier transport can enhance its immunogenicity and efficiency [90]. Currently, nucleic acid vectors, including viruses (influenzavirus, adenovirus, poliovirus, etc), fungi (*Saccharomyces*), and bacteria (*Bacillus Calmette Guerin*), among others, are commonly used. As carriers, BGs can be internalized by various cells, such as mouse macrophage raw 264.7, HCDECs, DCs, and Caco-2 [76,86,91]. In vitro cell experiments have shown that 60% of macrophages (raw 264.7) can internalize BGs containing reporter plasmids and then express green fluorescent proteins [92]. DCs can also internalize BGs and secrete IL-12 to activate Th1 immune responses [93]. The internalization and activation of BGs by APC cells provides a new strategy for vaccination and in situ immunotherapy. BGs carrying plasmids adopt the diffusion strategy (plasmid DNA diffuses through the lytic pore into the BGs) [92], and the nucleic acid can nonspecifically bind with BGs (The negatively charged DNA binds to positively charged groups in the inner membrane, such as amines). Each BG can load 4000–5000 copies of plasmid DNA (from medium to large plasmids) [94]. These in vitro experiments have proven that BGs are feasible for nucleic acid delivery. In mice models, there have been many studies of BG-based nucleic acid vaccines. Zhou et al. used BGs as a delivery system to prepare an effective DNA vaccine for the prevention of *C. psittaci* infection [79]. This BG-based DNA vaccine induced a stronger humoral immunity (IgG upregulation) and cellular immunity (Th1 type immune related indicator upregulation) than the naked DNA vaccine and BGs. Jiao et al. used the *Salmonella* ghost to prepare DNA vaccines to prevent *Neisseria gonorrhoea* [80]. They found that BG-based DNA vaccines induced stronger humoral immunities (IgG upregulation) and lymphocyte proliferation, relative to naked DNA vaccines and BGs alone. In a model experiment with bone marrow-derived DCs (BMDCs), Jiao et al. cultured the vaccine with BMDCs [95]. They found that the BG-based DNA vaccine promoted greater DCs maturation and activation (up regulation of cell surface costimulatory molecules CD80, CD86, CD40, and MHC-II) than the naked DNA vaccine and BGs alone. These results show that DNA vaccines prepared with BG-loaded plasmids have a better stimulatory effect on both humoral and cellular immunities, relative to naked DNA vaccines. Cao et al. used *DH5α* ghosts loaded with plasmids containing five exogenous fragments (including invariant chain-like protein [Iclp] gene) to prepare double-targeted DNA vaccines for oral immunization of grass carp [81]. Plasmids with endogenous Iclp easily enter the MHC-II antigen presentation pathway. Immunization with the double-targeted DNA vaccine substantially increases the activities of three innate immune parameters (SOD, LZM and C3) in serum and intestinal mucus. Moreover, the relative survival rate of the experimental group reached 81.11%, demonstrating the efficiency of the vaccine against *Vibrio* strains. These studies show that antigenic genes in BGs can be internalized and expressed by APCs, and foreign genes can be derived from multiple plasmids or a plasmid containing the fusion gene fragment. The multigene BGs vaccine induced stronger immune responses than the naked DNA vaccine.

### 4.2. Protein Antigen Vaccines

BGs serve as carriers for protein vaccines in two ways. (I) Multi epitope peptide BGs: through genetic engineering, the antigen and protein target is displayed on the surface to improve its immunogenicity and targeting. (II) Non-recombinant BGs mixture: when the antigen is co-incubated with BGs, the protein non-specifically binds the intima. However, to obtain strong immune responses, optimal doses of proteins and BGs should be investigated further. Tuntufye HN et al. found that BG-based recombinant vaccines of ferri-siderophore receptors protected chickens against avian pathogenic *E. coli* APEC infection [83]. They found that both the recombinant BGs vaccine and the non-recombinant mixture vaccine could significantly increase IgG immune responses and reduce mortality after infection. Moreover, the mortality rate for recombinant BGs chickens was lower, relative to that of the non-recombinant mixture vaccine. Sai Gong et al. developed a vaccine against hand-foot-and-mouth disease by expressing antigenic proteins of *Entero* virus *71* and *Coxsackie* virus in the outer membrane protein A(OMPA) of *E. coli* O157: H7 [15]. The vaccine increased IgG and IgA secretion, thereby inducing mucosal immunity. Moreover, the vaccine candidate protected mice against *E. coli* infection. In addition to expressing protein antigens on the outer membrane of BGs, Riedmann et al. enhanced cellular immune responses via intestinal or lung inoculation of BGs developed by fusing the *Haemophilus influenzae* (NTHi) antigen (OMP26) into the S layer or periplasmic space of *E. coli* [42]. In conclusion, endomembrane proteins, periplasmic space proteins and outer membrane proteins on BGs are antigenic, and they can induce immunogenicity. Compared to the protein subunit vaccine, BGs adjuvants significantly enhance the immunogenicity of antigenic proteins [20].

**Figure 5:**
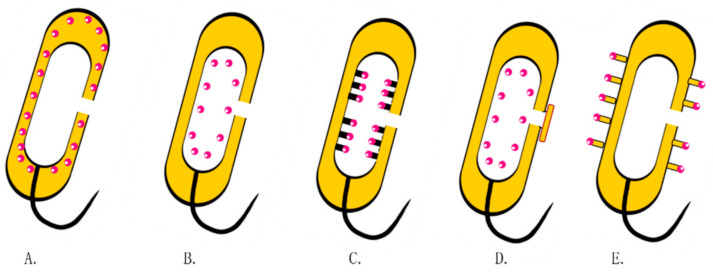
Drug loading strategies in BGs. (**A**) Expressions of proteins or antigens in the periplasmic space through genetic engineering. (**B**) Non-specific binding of drugs to the inner membrane. (**C**) Attachment of exogenous proteins or antigens onto the inner membrane using streptavidin. (**D**) BGs encapsulated with membranous vesicles. (**E**) Multiepitope peptide BGs.

Currently, there is increasing attention on how to improve the protein loading capacities of BGs (Figure 5). To achieve this, attempts have been made to express streptavidin in the inner membrane of BGs, whereas the target protein is phthalated with biotin. The specific interaction between biotin and streptavidin immobilizes the target protein in the inner membrane. In other studies, the protein was attached to the bacterial membrane through genetic engineering. For example, Sührer et al. attached galactosidase anchors on BGs containing cytochrome b5 to immobilize the enzyme [96]. In other studies, BGs have been sealed by fusions with membrane vesicles [97].

BGs can either be mixed with the antigen of interest, or the antigen can be expressed in a Gram-negative bacterium to be turned into BGs, thereby creating a bacterial shell with integrated antigens. Given the success of nucleic acid vaccines for COVID-19, BG-based vaccine research should fully utilize the natural intrinsic adjuvant effect of BGs in its vaccine candidates.

## 5. Drug Delivery Systems Based on BGs

### 5.1. BGs for Delivery of Nucleic Acids

BGs have potential as nucleic acid drug delivery carriers. For example, BGs’ analogue outer membrane vesicles (OMVs) have the ability to target macrophages [98]. SiRNA@OMVs targets macrophages in the tumor microenvironment, affecting the metabolic phenotype of macrophages by down regulating the redd1 gene of macrophages, thereby transforming macrophages into tumor suppressive types. This paper provides a reference for future studies on BG-based nucleic acid delivery systems for cancer treatment.

### 5.2. BGs Protein Delivery Systems

BGs loaded with protein drugs can be used in tumor immunotherapy. Kraśko et al. developed BGs from *E. coli* Nissle 1917(ECN) for transporting Lewis lung cancer cell lysates, including tumor associated antigens, to inhibit recurrence after primary resection [84]. Through mice models, we found that immunotherapy significantly enhanced the overall survival rate of experimental mice, circulation of CD8a + T cells, reduced metastasis incidences, and stimulated the immune system. Overall, this system has potential clinical applications in cancer treatment.

### 5.3. BGs for Delivery of Chemical Drugs

Targeting of BGs results from specific binding of the surface ligand to the recipient cell, and its surface adhesion factors contribute to adhesion of BGs to the target cell.

BGs play various roles in the treatment of bacterial infections. They stimulate innate immunity to enhance interactions between APCs and T cells, promoting inflammation [94]. Lim et al. delivered bedaquinoline and delamani, second-line tuberculosis drugs, to the lungs of mice via *Mycobacterium bovis* BGs [88]. The drugs effectively killed the bacteria and significantly increased the number of innate immune cells (neutrophils, macrophages, and DCs), adaptive immune cells (CD4 effector cells), and levels of Th1 cytokines (TNFα, IFNγ and IL-12p70). It also stimulated NO production by macrophages to kill the bacteria. Xie et al. killed bacteria in macrophages by delivering ciprofloxacin (CIP) via ECN ghosts [89]. ECN ghosts have high loading capacities and high targeting efficiencies. ECN ghosts showed that the loading of CIP was as high as 12.5%, which accumulated in the liver and spleen after intravenous injection. Specifically, after targeting to macrophages, CIP@ECN ghosts promote the release of intracellular lysozyme and secretion of pro-inflammatory cytokines (TNF-α and IL-6), thereby destroying bacteria in macrophages without any obvious side effects. Bactericidal therapy caused by BGs provides the possibility of alternative antibiotic therapy.

BGs can also be used as chemotherapy drug carriers for tumor immunotherapy. BGs that are specific to cancer cells, such as ECN ghost, have been developed [99]. Experiments on several in vitro tumor cells (hepatoma carcinoma cell and colorectal adenocarcinoma cells) showed that chemotherapeutics@BGs could adhere to tumor cells and exhibited stronger cytotoxicities than free chemotherapeutics, thereby reducing the doses of chemotherapeutic drugs [85,87,100]. Chemotherapeutics@BGs can achieve immunotherapy against tumors in animal experiments. Groza et al. reported that delivery of oxaliplatin via BGs exerted stronger anticancer activities against CT26 cell allografts [82]. In rat models, drug delivery through BGs significantly increased the survival rate of mice with colorectal cancer, which was attributed to enhanced anti-tumor memory effects. Accumulation of BGs in tumors may result from local deletion of the immune system in the tumor microenvironment. This deletion inhibits BG clearance from the tumor area. Some living bacteria, such as ECN, have natural tumor microenvironment targeting properties. Songzhi Xie et al. loaded the chemotherapeutic drug (5-Fu) and the macrophage phenotype regulator zoledronic acid (ZOL) into ECN through electroporation, and modified Au nanorods on the surface [5]. When ECN was targeted and colonized into the intestinal tumor area, it was irradiated by near infrared (NIR) to convert ECN into ECN ghosts through temperature control induction, and the drug was released from the pore. Finally, macrophages can effectively become polarized to produce proinflammatory cytokines and inhibit tumor growth. They combined immunotherapy and photothermal effects to treat cancer. Given the tumor microenvironment, it is more flexible to use environment-induced promoters such as hypoxic promoters and temperature-controlled promoters to induce site-specific lysis of tumor-targeted bacteria. Overall, BG-based delivery systems are potential chemotherapeutic strategies [88].

## 6. Drug Delivery Route for BGs

**Figure 6:**
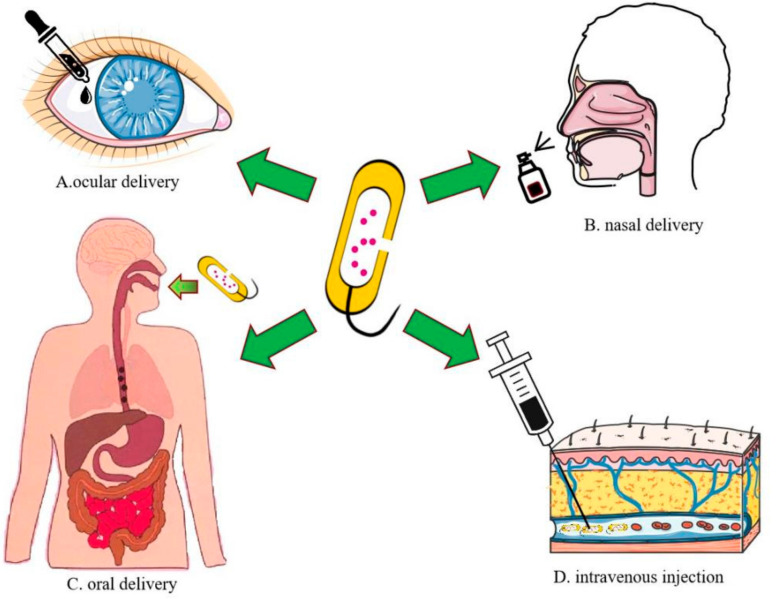
Route of administration of BGs.

As a natural bioactive delivery system, BGs can be administered in a variety of ways, including mucosal administration (nasal cavity, gastrointestinal tract, and ocular administration) and intravenous injection (Figure 6) [11]. Although there is an example of intraperitoneal injection of BGs in mice to treat colon cancer, mucosal administration is associated with enhanced treatment adherence [82]. In the co-culture experiment of BGs and Caco-2, BGs adhered to these cells, proving the existence of adhesion related factors on BGs’ surfaces. The presence of TLR4/TLR5 on epithelial cells laid the foundation for the use of BGs as mucosal vaccines. In addition, in one study, the mucosal route induced immune responses, which was comparable, or even better than the intra injection route [101]. Inic Kanada et al. used BGs to prepare multi-epitope peptides to prevent trachoma in mice and guinea pigs, and the specific IgA level was significantly increased, compared to subcutaneous administration [102]. Therefore, mucosal administration is a promising method. Ocular surface delivery using BGs as a carrier is only used to prevent trachoma infected by mycoplasmas [103]. In addition, the oral route of mucosal administration is highly safe, convenient, and widely acceptable [104]. Oral administration of BGs has been tested in fish [105]. BGs have certain oral tolerance thresholds, and they can be used as carriers for oral delivery, especially probiotics. A recent study evaluated the treatment of colon cancer by inducing lysis to produce BGs after oral administration of probiotic ECN in mice [5]. Preclinical evidence suggests that bacterial adherence to host tissues and regulation of natural immunity may be unrelated to bacterial activities [106]. Killed probiotics have certain anti-inflammatory effects [107]. Moreover, oral administration of dead *Akkermansia* lowers lipids levels in blood [108]. These findings prove that BG-based oral probiotics delivery systems may help alleviate inflammatory bowel disease (IBD) and obesity. Finally, human clinical research should be conducted to prove the safety and efficacy of BG-based treatment strategies, and explore individualized treatment programs.

## 7. Conclusions and Perspectives

BGs are a new drug delivery system with many biological characteristics. BGs can easily load nucleic acids, proteins and chemicals; therefore, they are suitable for mass production. Since BGs do not contain genetic material, there is no risk for horizontal gene transfer. Given that BGs are bacterial cell wall enclosures, they express and can be loaded with multiple antigens or drugs, which can enhance immunogenicity or treatment efficacies of drugs. BGs can target specific cells, such as APCs, HCDEC, and Caco-2, among others. The immunogenicity and targeting of BGs can be used for tumor immunotherapy and vaccines. In addition, the BGs probiotic system has great potential as an oral system for intestinal diseases (e.g., colon cancer, IBD). Oral administration reduces the efficacy of many drugs. BG-based delivery systems have the potential to override this limitation.

However, various challenges are associated with clinical applications of BGs. For instance, therapeutic effects of BGs of different strains combined with antigens or drugs should be investigated. Moreover, leaking pores of BGs should be sealed, and the stability as well as mechanisms of the combination of various drugs or antigens with BGs should also be investigated. BGs can easily be cleared by the immune system in vivo, therefore, it is important to improve their targeting and stability. Recent BG-based studies have focused on designs and combined applications of BGs with additional novel features, such as tumor targeted ECN ghosts. Combined with photothermal therapy, tumor immunotherapy is expected to solve the poor sealing of BGs and realize the function of targeted drug release to inhibit tumors. Through surface modifications and genetic engineering, BGs have the potential to become powerful delivery vehicles for drugs or vaccines.

## Figures and Tables

**Table 1:** Application strategies of BGs-based delivery systems.

Lysis Plasmid	Contents	Strain Name of BGs	Application	Reference	Model
/	pcDNA of Macrophage infectivity potentiator (MIP) and pcDNA of *C. psittaci* major outer membrane protein (MOMP)	*E. coli JM109*	DNA vaccine for Preventing *C. psittaci* infection	[79]	mice
pBBR1MCS-E	Recombinant plasmid pVAX1-nspA	*S.enteritidis*	DNA vaccine for Preventing *Gonococcal*	[80]	mice
pBV220-E	DNA of *Vibrio mimicus* epitopes OmpU and VMH in tandem	*E. coli DH5α*	Oral DNA vaccine for Preventing *Vibrio mimicus* infection	[81]	grass carps
/	Oxaliplatin	ECN	Injection of oxaliplatin@BGs to induce immunogenic cell death (ICD) for therapy of colon cancer	[82]	mice
pML1, pDKL01	NTHi antigen OMP26	*E. coli* pop2135	Mucosal delivery of OMP26@BGs for immunization against NTHi infection	[42]	rat
pBAD-E	Ferri-siderophore receptors FepA, FhuE, IroN and IutA	*E. coli APEC*	Recombinant BGs vaccine mucosal vaccine to prevent APEC infection	[83]	chicken
/	Lewis tumour cell lysate	ECN	BGs carries tumor lysates to enhance tumor immunogenicity, induce immune cells to mature and attack cancer cells	[84]	mice
pBV-mELS	Epothilone B	ECN	Epothilone B@BGs as an anticancer drug to treat cancer	[85]	HeLa cell
pSON1	DOX	*Mannheimia haemolytica*	DOX@BGs is targeted to human colon adenocarcinoma cells for the treatment of cancer	[86]	Caco-2
pLysS	5-FU	*E. coli* BL21 (DE3)	5-FU@BGs treatment of various types of colorectal cancer	[87]	Caco-2
/	Bedaquiline and delamanid	*Mycobacterium tuberculosis*	Bedaquiline/delamanid@BGs stimulate the immune system to kill bacteria and treat tuberculosis	[88]	mice
pBV220-E	CIP	ECN	CIP@BGs is used to destroy Staphylococcus aureus in macrophages	[89]	macrophage RAW264.7/mice
pBV220-E	ZOL/5-FU	ECN	ZOL/5-FU@BGs promote macrophage polarization and kill tumor	[5]	mice

## Data Availability

Not applicable.

## Abbreviations

BGs	Bacterial ghosts
MraY	Phospho-MurNAc-pentapeptide translocase
BPL	β-propiolactone
DAP	diaminopimelic acid
SNUC	staphylococcal nuclease
MAP	model amphipathic peptide
PAMPs	pathogen-associated molecular patterns
LPS	lipopolysaccharide
LPP	lipoprotein
TLR4	*Toll*-like receptor 4
IFN-1	I interferon
MIC	minimum inhibitory concentration
TLR2	*Toll*-like receptor 2
APCs	antigen-presenting cells
NLRC4	*Nod*-like receptor card domain containing 4
PGN	peptidoglycan
HCDECs	human conjunctiva-derived epithelial cells
BMDCs	bone marrow-derived DCs
Iclp	invariant chain-like protein
IBD	inflammatory bowel disease
OMVs	outer membrane vesicles
OMPA	outer membrane protein A
CIP	ciprofloxacin
ECN *E*	*coli* nissle 1917
5-FU	5-fluorouridine
DC	dendritic cell

## References

[1] Peng R., Ji H., Jin L., Lin S., Huang Y., Xu K., Yang Q., Sun D., Wu W. (2020). Macrophage-Based Therapies for Atherosclerosis Management. J. Immunol. Res..

[2] Sun D., Chen J., Wang Y., Ji H., Peng R., Jin L., Wu W. (2019). Advances in Refunctionalization of Erythrocyte-Based Nanomedicine for Enhancing Cancer-Targeted Drug Delivery. Theranostics.

[3] Moghimipour E., Abedishirehjin S., Baghbadorani M.A., Handali S. (2021). Bacteria and Archaea: A New Era of Cancer Therapy. J. Control. Release.

[4] Ding C., Cicuttini F., Li J., Jones G. (2009). Targeting IL-6 in the Treatment of Inflammatory and Autoimmune Diseases. Expert Opin. Investig. Drugs.

[5] Xie S., Zhang P., Zhang Z., Liu Y., Chen M., Li S., Li X. (2021). Bacterial Navigation for Tumor Targeting and Photothermally-Triggered Bacterial Ghost Transformation for Spatiotemporal Drug Release. Acta Biomater..

[6] Holay M., Guo Z., Pihl J., Heo J., Park J.H., Fang R.H., Zhang L. (2021). Bacteria-Inspired Nanomedicine. ACS Appl. Bio Mater..

[7] Hosseinidoust Z., Mostaghaci B., Yasa O., Park B.-W., Singh A.V., Sitti M. (2016). Bioengineered and Biohybrid Bacteria-Based Systems for Drug Delivery. Adv. Drug Deliv. Rev..

[8] Senevirathne A., Hewawaduge C., Lee J.H. (2021). Immunization of Chicken with Flagellin Adjuvanted *Salmonella enteritidis* Bacterial Ghosts Confers Complete Protection against Chicken Salmonellosis. Poult. Sci..

[9] Carvalho S.B., Peixoto C., Carrondo M.J.T., Silva R.J.S. (2021). Downstream Processing for Influenza Vaccines and Candidates: An Update. Biotechnol. Bioeng..

[10] Sabbaghi A., Miri S.M., Keshavarz M., Zargar M., Ghaemi A. (2019). Inactivation Methods for Whole Influenza Vaccine Production. Rev. Med. Virol..

[11] Sheweita S.A., Batah A.M., Ghazy A.A., Hussein A., Amara A.A. (2019). A New Strain of *Acinetobacter Baumannii* and Characterization of Its Ghost as a Candidate Vaccine. J. Infect. Public Health.

[12] Michalek J., Hezova R., Turanek-Knötigova P., Gabkova J., Strioga M., Lubitz W., Kudela P. (2017). Oncolysate-Loaded *Escherichia Coli* Bacterial Ghosts Enhance the Stimulatory Capacity of Human Dendritic Cells. Cancer Immunol. Immunother..

[13] Wang G., Zhao G., Chao X., Xie L., Wang H. (2020). The Characteristic of Virulence, Biofilm and Antibiotic Resistance of *Klebsiella pneumoniae*. Int. J. Environ. Res. Public Health.

[14] Muhammad A., Champeimont J., Mayr U.B., Lubitz W., Kudela P. (2012). Bacterial Ghosts as Carriers of Protein Subunit and DNA-Encoded Antigens for Vaccine Applications. Expert Rev. Vaccines.

[15] Gong S., Nan N., Sun Y., He Z., Li J., Chen F., Li T., Ning N., Wang J., Li Z. (2020). Protective Immunity Elicited by VP1 Chimeric Antigens of Bacterial Ghosts against Hand-Foot-and-Mouth Disease Virus. Vaccines.

[16] Batah A.M., Ahmad T.A. (2020). The Development of Ghost Vaccines Trials. Expert Rev Vaccines.

[17] Ganeshpurkar A., Ganeshpurkar A., Pandey V., Agnihotri A., Bansal D., Dubey N. (2014). Harnessing the Potential of Bacterial Ghost for the Effective Delivery of Drugs and Biotherapeutics. Int. J. Pharm. Investig..

[18] Li Z., Wang Y., Liu J., Rawding P., Bu J., Hong S., Hu Q. (2021). Chemically and Biologically Engineered Bacteria-Based Delivery Systems for Emerging Diagnosis and Advanced Therapy. Adv. Mater..

[19] Mendel S., Holbourn J.M., Schouten J.A., Bugg T.D.H. (2006). Interaction of the Transmembrane Domain of Lysis Protein E from Bacteriophage ΦX174 with Bacterial Translocase MraY and Peptidyl-Prolyl Isomerase SlyD. Microbiology.

[20] Langemann T., Koller V.J., Muhammad A., Kudela P., Mayr U.B., Lubitz W. (2010). The Bacterial Ghost Platform System: Production and Applications. Bioeng. Bugs.

[21] Lubitz P., Mayr U.B., Lubitz W., Guzmán C.A., Feuerstein G.Z. (2009). Applications of Bacterial Ghosts in Biomedicine. Pharmaceutical Biotechnology.

[22] Chung T.-C., Jones C.H., Gollakota A., Kamal Ahmadi M., Rane S., Zhang G., Pfeifer B.A. (2015). Improved *Escherichia Coli* Bactofection and Cytotoxicity by Heterologous Expression of Bacteriophage ΦX174 Lysis Gene E. Mol. Pharm..

[23] Szostak M.P., Hensel A., Eko F.O., Klein R., Auer T., Mader H., Haslberger A., Bunka S., Wanner G., Lubitz W. (1996). Bacterial Ghosts: Non-Living Candidate Vaccines. J. Biotechnol..

[24] Witte A., Wanner G., Sulzner M., Lubitz W. (1992). Dynamics of PhiX174 Protein E-Mediated Lysis of *Escherichia Coli*. Arch. Microbiol..

[25] Haeusser D.P., Margolin W. (2016). Splitsville: Structural and Functional Insights into the Dynamic Bacterial Z Ring. Nat. Rev. Microbiol..

[26] Witte A., Brand E., Mayrhofer P., Narendja F., Lubitz W. (1998). Mutations in Cell Division Proteins FtsZ and FtsA Inhibit ΦX174 Protein-E-Mediated Lysis of *Escherichia Coli*. Arch. Microbiol..

[27] Marchart J., Dropmann G., Lechleitner S., Schlapp T., Wanner G., Szostak M.P., Lubitz W. (2003). *Pasteurella Multocida*- and *Pasteurella Haemolytica*-Ghosts: New Vaccine Candidates. Vaccine.

[28] Jalava K. (2002). Bacterial Ghosts as Vaccine Candidates for Veterinary Applications. J. Control. Release.

[29] Hajam I.A., Dar P.A., Won G., Lee J.H. (2017). Bacterial Ghosts as Adjuvants: Mechanisms and Potential. Vet. Res..

[30] Paukner S., Stiedl T., Kudela P., Bizik J., Al Laham F., Lubitz W. (2006). Bacterial Ghosts as a Novel Advanced Targeting System for Drug and DNA Delivery. Expert Opin. Drug Deliv..

[31] Barisani-Asenbauer T., Montanaro J., Inic-Kanada A., Ladurner A., Stein E., Belij S., Bintner N., Schlacher S., Schuerer N., Mayr U.B. (2015). *Escherichia Coli* Nissle 1917 Bacterial Ghosts Retain Crucial Surface Properties and Express Chlamydial Antigen: An Imaging Study of a Delivery System for the Ocular Surface. DDDT.

[32] Farjadian F., Moghoofei M., Mirkiani S., Ghasemi A., Rabiee N., Hadifar S., Beyzavi A., Karimi M., Hamblin M.R. (2018). Bacterial Components as Naturally Inspired Nano-Carriers for Drug/Gene Delivery and Immunization: Set the Bugs to Work?. Biotechnol. Adv..

[33] Koller V.J., Dirsch V.M., Beres H., Donath O., Reznicek G., Lubitz W., Kudela P. (2013). Modulation of Bacterial Ghosts—Induced Nitric Oxide Production in Macrophages by Bacterial Ghost-Delivered Resveratrol. FEBS J.

[34] Zhu W., Zhang Y., Liu X. (2015). Efficient Production of Safety-Enhanced *Escherichia Coli* Ghosts by Tandem Expression of PhiX 174 Mutant Gene E and *Staphylococcal* Nuclease A Gene. Microbiol. Res..

[35] Tian Q., Zhou W., Si W., Yi F., Hua X., Yue M., Chen L., Liu S., Yu S. (2018). Construction of *Salmonella Pullorum* Ghost by Co-Expression of Lysis Gene E and the Antimicrobial Peptide SMAP29 and Evaluation of Its Immune Efficacy in Specific-Pathogen-Free Chicks. J. Integr. Agric..

[36] Hjelm A., Söderström B., Vikström D., Jong W.S.P., Luirink J., de Gier J.-W. (2015). Autotransporter-Based Antigen Display in Bacterial Ghosts. Appl. Environ. Microbiol..

[37] Senevirathne A., Hewawaduge C., Park J.-Y., Park S., Lee J.H. (2020). Parenteral Immunization of *Salmonella Typhimurium* Ghosts with Surface-Displayed *Escherichia Coli* Flagellin EnhancesTLR-5 Mediated Activation of Immune Responses That Protect the Chicken against *Salmonella* Infection. Microb. Pathog..

[38] Ekong E.E., Okenu D.N., Mania-Pramanik J., He Q., Igietseme J.U., Ananaba G.A., Lyn D., Black C., Eko F.O. (2009). A *Vibrio Cholerae* Ghost-Based Subunit Vaccine Induces Cross-Protective Chlamydial Immunity That Is Enhanced by CTA2B, the Nontoxic Derivative of Cholera Toxin. FEMS Immunol. Med Microbiol..

[39] Hatfaludi T., Liska M., Zellinger D., Ousman J.P., Szostak M., Jalava K., Lubitz W. (2004). Bacterial Ghost Technology for Pesticide Delivery. J. Agric. Food Chem..

[40] Chen J., Li N., She F. (2014). *Helicobacter Pylori* Outer Inflammatory Protein DNA Vaccine-Loaded Bacterial Ghost Enhances Immune Protective Efficacy in C57BL/6 Mice. Vaccine.

[41] Muhammad A., Kassmannhuber J., Rauscher M., Falcon A.A., Wheeler D.W., Zhang A.A., Lubitz P., Lubitz W. (2019). Subcutaneous Immunization of Dogs With *Bordetella bronchiseptica* Bacterial Ghost Vaccine. Front. Immunol..

[42] Riedmann E.M., Lubitz W., McGrath J., Kyd J.M., Cripps A.W. (2011). Effectiveness of Engineering the Nontypeable *Haemophilus Influenzae* Antigen Omp26 as an S-Layer Fusion in Bacterial Ghosts as a Mucosal Vaccine Delivery. Hum. Vaccines.

[43] Ran X., Meng X.-Z., Geng H.-L., Chang C., Chen X., Wen X., Ni H. (2019). Generation of Porcine *Pasteurella Multocida* Ghost Vaccine and Examination of Its Immunogenicity against Virulent Challenge in Mice. Microb. Pathog..

[44] Wang S., Li Z., Zhang J., Xi L., Cui Y., Zhang W., Zhang J., Zhang H. (2020). A Safe Non-Toxic *Brucella Abortus* Ghosts Induce Immune Responses and Confer Protection in BALB/c Mice. Mol. Immunol..

[45] Jiang N., Luo L., Xing W., Li T., Yuan D., Xu G., Li W., Ma Z., Jin L., Ji M. (2019). Generation and Immunity Effect Evaluation of Biotechnology-Derived *Aeromonas Veronii* Ghost by PhiX174 Gene E-Mediated Inactivation in Koi (*Cyprinus Carprio Koi*). Fish Shellfish Immunol..

[46] Panoff J.M., Chuiton C. (2004). Horizontal Gene Transfer: A Universal Phenomenon. Hum. Ecol. Risk Assess..

[47] Soleymani S., Tavassoli A., Hashemi Tabar G., Kalidari G.A., Dehghani H. (2020). Design, Development, and Evaluation of the Efficacy of a Nucleic Acid-Free Version of a Bacterial Ghost Candidate Vaccine against Avian Pathogenic *E. Coli* (APEC) O78:K80 Serotype. Vet. Res..

[48] Ronchel M.C., Molina L., Witte A., Lutbiz W., Molin S., Ramos J.L., Ramos C. (1998). Characterization of Cell Lysis in Pseudomonas Putida Induced upon Expression of Heterologous Killing Genes. Appl. Environ. Microbiol..

[49] Kloos D.U., Strätz M., Güttler A., Steffan R.J., Timmis K.N. (1994). Inducible Cell Lysis System for the Study of Natural Transformation and Environmental Fate of DNA Released by Cell Death. J. Bacteriol..

[50] Amara A.A., Salem-Bekhit M.M., Alanazi F.K. (2013). Sponge-Like: A New Protocol for Preparing Bacterial Ghosts. Sci. World J..

[51] Rabea S., Salem-Bekhit M.M., Alanazi F.K., Yassin A.S., Moneib N.A., Hashem A.E.M. (2018). A Novel Protocol for Bacterial Ghosts’ Preparation Using Tween 80. Saudi Pharm. J..

[52] Hu J., Dong H., Fu L., Zuo J., Hu S. (2017). Comparison of Three Methods for Preparation of Bacterial Ghosts from Avian Pathogenic *Escherichia Coli*. Sheng Wu Gong Cheng Xue Bao = Chin. J. Biotechnol..

[53] Palm-Apergi C., Hällbrink M. (2008). A New Rapid Cell-Penetrating Peptide Based Strategy to Produce Bacterial Ghosts for Plasmid Delivery. J. Control. Release.

[54] Yu S., Peng W., Si W., Yin L., Liu S., Liu H., Zhao H., Wang C., Chang Y., Lin Y. (2011). Enhancement of Bacteriolysis of Shuffled Phage PhiX174 Gene E. Virol. J..

[55] Vinod N., Oh S., Park H.J., Koo J.M., Choi C.W., Kim S.C. (2015). Generation of a Novel *Staphylococcus Aureus* Ghost Vaccine and Examination of Its Immunogenicity against Virulent Challenge in Rats. Infect. Immun..

[56] Dobrovolskaia M.A. (2017). Dendrimers Effects on the Immune System: Insights into Toxicity and Therapeutic Utility. CPD.

[57] Burns K., Martinon F., Esslinger C., Pahl H., Schneider P., Bodmer J.-L., Di Marco F., French L., Tschopp J. (1998). MyD88, an Adapter Protein Involved in Interleukin-1 Signaling. J. Biol. Chem..

[58] Piras V., Selvarajoo K. (2014). Beyond MyD88 and TRIF Pathways in Toll-Like Receptor Signaling. Front. Immunol..

[59] Tough D.F., Sun S., Sprent J. (1997). T Cell Stimulation In Vivo by Lipopolysaccharide (LPS). J. Exp. Med..

[60] Zhang X., Qi C., Guo Y., Zhou W., Zhang Y. (2016). Toll-like Receptor 4-Related Immunostimulatory Polysaccharides: Primary Structure, Activity Relationships, and Possible Interaction Models. Carbohydr. Polym..

[61] Brown J., Wang H., Hajishengallis G.N., Martin M. (2011). TLR-Signaling Networks: An Integration of Adaptor Molecules, Kinases, and Cross-Talk. J. Dent. Res..

[62] Fitzgerald K.A., Kagan J.C. (2020). Toll-like Receptors and the Control of Immunity. Cell.

[63] Xia P., Wu Y., Lian S., Yan L., Meng X., Duan Q., Zhu G. (2021). Research Progress on Toll-like Receptor Signal Transduction and Its Roles in Antimicrobial Immune Responses. Appl. Microbiol. Biotechnol..

[64] McAleer J.P., Vella A.T. (2010). Educating CD4 T Cells with Vaccine Adjuvants: Lessons from Lipopolysaccharide. Trends Immunol..

[65] Razim A., Pacyga K., Naporowski P., Martynowski D., Szuba A., Gamian A., Górska S. (2021). Identification of Linear Epitopes on the Flagellar Proteins of Clostridioides Difficile. Sci. Rep..

[66] Gries C.M., Mohan R.R., Morikis D., Lo D.D. (2019). Crosslinked Flagella as a Stabilized Vaccine Adjuvant Scaffold. BMC Biotechnol..

[67] Ramos H.C., Rumbo M., Sirard J.C. (2004). Bacterial Flagellins: Mediators of Pathogenicity and Host Immune Responses in Mucosa. Trends Microbiol..

[68] Wen J., Xuan B., Liu Y., Wang L., He L., Meng X., Zhou T., Wang Y. (2021). Updating the NLRC4 Inflammasome: From Bacterial Infections to Autoimmunity and Cancer. Front. Immunol..

[69] Yu H., Zhou X., Wen S., Xiao Q. (2012). Flagellin/TLR5 Responses Induce Mucus Hypersecretion by Activating EGFR via an Epithelial Cell Signaling Cascades. Exp. Cell Res..

[70] Miao E.A., Andersen-Nissen E., Warren S.E., Aderem A. (2007). TLR5 and Ipaf: Dual Sensors of Bacterial Flagellin in the Innate Immune System. Semin. Immunopathol..

[71] Buwitt-Beckmann U., Heine H., Wiesmuller K.-H., Jung G., Brock R., Ulmer A.J. (2005). Lipopeptide Structure Determines TLR2 Dependent Cell Activation Level. FEBS J..

[72] Hug H., Mohajeri M., La Fata G. (2018). Toll-Like Receptors: Regulators of the Immune Response in the Human Gut. Nutrients.

[73] Warshakoon H.J., Hood J.D., Kimbrell M.R., Malladi S., Wu W.Y., Shukla N., Agnihotri G., Sil D., David S.A. (2009). Potential Adjuvantic Properties of Innate Immune Stimuli. Hum. Vaccines.

[74] Carneiro L.a.M., Travassos L.H., Philpott D.J. (2004). Innate Immune Recognition of Microbes through Nod1 and Nod2: Implications for Disease. Microbes Infect..

[75] Chandler C.E., Ernst R.K. (2017). Bacterial Lipids: Powerful Modifiers of the Innate Immune Response. F1000Research.

[76] Kudela P., Koller V.J., Mayr U.B., Nepp J., Lubitz W., Barisani-Asenbauer T. (2011). Bacterial Ghosts as Antigen and Drug Delivery System for Ocular Surface Diseases: Effective Internalization of Bacterial Ghosts by Human Conjunctival Epithelial Cells. J. Biotechnol..

[77] Trombetta E.S., Mellman I. (2005). Cell Biology of Antigen Processing In Vitro and In Vivo. Annu. Rev. Immunol..

[78] Felnerova D., Kudela P., Bizik J., Haslberger A., Lubitz W. (2004). T Cell-Specific Immune Response Induced by Bacterial Ghosts. Med. Sci. Monit..

[79] Zhou P., Wu H., Chen S., Bai Q., Chen X., Chen L., Zeng X., Liu L., Chen L. (2019). MOMP and MIP DNA-Loaded Bacterial Ghosts Reduce the Severity of Lung Lesions in Mice after Chlamydia Psittaci Respiratory Tract Infection. Immunobiology.

[80] Jiao H., Yang H., Zheng W., Zhang Q., Zhao D., Li G. (2020). Enhancement of Immune Responses by Co-administration of Bacterial Ghosts-mediated *Neisseria Gonorrhoeae* DNA Vaccines. J. Appl. Microbiol..

[81] Cao J., Zhu X.-C., Liu X.-Y., Yuan K., Zhang J.-J., Gao H.-H., Li J.-N. (2019). An Oral Double-Targeted DNA Vaccine Induces Systemic and Intestinal Mucosal Immune Responses and Confers High Protection against *Vibrio Mimicus* in Grass Carps. Aquaculture.

[82] Groza D., Gehrig S., Kudela P., Holcmann M., Pirker C., Dinhof C., Schueffl H.H., Sramko M., Hoebart J., Alioglu F. (2018). Bacterial Ghosts as Adjuvant to Oxaliplatin Chemotherapy in Colorectal Carcinomatosis. OncoImmunology.

[83] Tuntufye H.N., Ons E., Pham A.D.N., Luyten T., Van Gerven N., Bleyen N., Goddeeris B.M. (2012). *Escherichia Coli* Ghosts or Live *E. coli* Expressing the Ferri-Siderophore Receptors FepA, FhuE, IroN and IutA Do Not Protect Broiler Chickens against Avian Pathogenic *E. Coli* (APEC). Vet. Microbiol..

[84] Kraśko J.A., Žilionytė K., Darinskas A., Strioga M., Rjabceva S., Zalutsky I., Derevyanko M., Kulchitsky V., Lubitz W., Kudela P. (2017). Bacterial Ghosts as Adjuvants in Syngeneic Tumour Cell Lysate-Based Anticancer Vaccination in a Murine Lung Carcinoma Model. Oncol. Rep..

[85] Zhu W., Hao L., Liu X., Borrás-Hidalgo O., Zhang Y. (2018). Enhanced Anti-Proliferative Efficacy of Epothilone B Loaded with *Escherichia Coli* Nissle 1917 Bacterial Ghosts on the HeLa Cells by Mitochondrial Pathway of Apoptosis. Drug Dev. Ind. Pharm..

[86] Paukner S., Kohl G., Lubitz W. (2004). Bacterial Ghosts as Novel Advanced Drug Delivery Systems: Antiproliferative Activity of Loaded Doxorubicin in Human Caco-2 Cells. J. Control. Release.

[87] Youssof A.M.E., Alanazi F.K., Salem-Bekhit M.M., Shakeel F., Haq N. (2019). Bacterial Ghosts Carrying 5-Fluorouracil: A Novel Biological Carrier for Targeting Colorectal Cancer. AAPS PharmSciTech.

[88] Alonso S. (2019). Harnessing the Immunomodulatory Properties of Bacterial Ghosts to Boost the Anti-Mycobacterial Protective Immunity. Front. Immunol..

[89] Xie S., Li S., Zhang Z., Chen M., Ran P., Li X. (2020). Bacterial Ghosts for Targeting Delivery and Subsequent Responsive Release of T Ciprofloxacin to Destruct Intracellular Bacteria. Chem. Eng. J..

[90] Xu Y., Pak-Wai Y., Jenny L. (2014). Intranasal DNA Vaccine for Protection against Respiratory Infectious Diseases: The Delivery Perspectives. Pharmaceutics.

[91] Stein E., Inic-Kanada A., Belij S., Montanaro J., Bintner N., Schlacher S., Mayr U.B., Lubitz W., Stojanovic M., Najdenski H. (2013). In Vitro and In Vivo Uptake Study of *Escherichia Coli* Nissle 1917 Bacterial Ghosts: Cell-Based Delivery System to Target Ocular Surface Diseases. Invest. Ophthalmol. Vis. Sci..

[92] Kudela P., Paukner S., Mayr U.B., Cholujova D., Schwarczova Z., Sedlak J., Bizik J., Lubitz W. (2005). Bacterial Ghosts as Novel Efficient Targeting Vehicles for DNA Delivery to the Human Monocyte-Derived Dendritic Cells. J. Immunother..

[93] Haslberger A.G., Kohl G., Felnerova D., Mayr U.B., Fürst-Ladani S., Lubitz W. (2000). Activation, Stimulation and Uptake of Bacterial Ghosts in Antigen Presenting Cells. J. Biotechnol..

[94] Walcher P., Mayr U.B., Azimpour-Tabrizi C., Eko F.O., Jechlinger W., Mayrhofer P., Alefantis T., Mujer C.V., DelVecchio V.G., Lubitz W. (2004). Antigen Discovery and Delivery of Subunit Vaccines by Nonliving Bacterial Ghost Vectors. Expert Rev. Vaccines.

[95] Jiao H., Yang H., Zhao D., Chen J., Zhang Q., Liang J., Yin Y., Kong G., Li G. (2018). Design and Immune Characterization of a Novel *Neisseria Gonorrhoeae* DNA Vaccine Using Bacterial Ghosts as Vector and Adjuvant. Vaccine.

[96] Sührer I., Langemann T., Lubitz W., Weuster-Botz D., Castiglione K. (2015). A Novel One-Step Expression and Immobilization Method for the Production of Biocatalytic Preparations. Microb Cell Fact.

[97] Paukner S., Kohl G., Jalava K., Lubitz W. (2003). Sealed Bacterial Ghosts—Novel Targeting Vehicles for Advanced Drug Delivery of Water-Soluble Substances. J. Drug Target..

[98] Guo Q., Li X., Zhou W., Chu Y., Chen Q., Zhang Y., Li C., Chen H., Liu P., Zhao Z. (2021). Sequentially Triggered Bacterial Outer Membrane Vesicles for Macrophage Metabolism Modulation and Tumor Metastasis Suppression. ACS Nano.

[99] Ou B., Yang Y., Tham W.L., Chen L., Guo J., Zhu G. (2016). Genetic Engineering of Probiotic *Escherichia Coli* Nissle 1917 for Clinical Application. Appl. Microbiol. Biotechnol..

[100] Rabea S., Alanazi F.K., Ashour A.E., Salem-Bekhit M.M., Yassin A.S., Moneib N.A., Hashem A.E.M., Haq N. (2020). Salmonella-Innovative Targeting Carrier: Loading with Doxorubicin for Cancer Treatment. Saudi Pharm. J..

[101] Wang Y., Li M., Sun Y., Qiu H.J. (2019). Microfold Cells-Targeting Antigen Delivery: A Promising Strategy to Enhance the Efficacy of Mucosal Vaccines. Chin. J. Biotechnol..

[102] Aleksandra I.K., Marijana S., Simone S., Elisabeth S., Sandra B.R., Emilija M., Ivana L., Jacqueline M., Nadine S., Nora B. (2015). Delivery of a Chlamydial Adhesin N-PmpC Subunit Vaccine to the Ocular Mucosa Using Particulate Carriers. PLoS ONE.

[103] Eko F.O., Barisani-Asenbauer T. (2008). Development of a *Chlamydia Trachomatis* Bacterial Ghost Vaccine to Fight Human Blindness. Hum. Vaccines.

[104] Zhang X., Han Y., Huang W., Jin M., Gao Z. (2021). The Influence of the Gut Microbiota on the Bioavailability of Oral Drugs. Acta Pharm. Sin. B.

[105] Ji S., Gong Q., Zhang W., Zheng J., Peng B., Yang M. (2020). Recombinant *Vibrio Parahaemolyticus* Ghosts Protect Zebrafish against Infection by *Vibrio* Species. Fish Shellfish Immunol..

[106] Sarkar S. (2018). Whether Viable and Dead Probiotic Are Equally Efficacious?. NFS.

[107] Lahtinen S.J. (2012). Probiotic Viability—Does It Matter?. Microb. Ecol. Health Dis..

[108] Plovier H., Everard A., Druart C., Depommier C., Van Hul M., Geurts L., Chilloux J., Ottman N., Duparc T., Lichtenstein L. (2017). A Purified Membrane Protein from *sAkkermansia Muciniphila* or the *Pasteurized Bacterium* Improves Metabolism in Obese and Diabetic Mice. Nat. Med..

